# A Strategic Synthesis of Orange Waste-Derived Porous Carbon via a Freeze-Drying Method: Morphological Characterization and Cytocompatibility Evaluation

**DOI:** 10.3390/molecules29163967

**Published:** 2024-08-22

**Authors:** Angela S. Kaloudi, Panagiota Zygouri, Konstantinos Spyrou, Antrea-Maria Athinodorou, Eirini Papanikolaou, Mohammed Subrati, Dimitrios Moschovas, K. K. R. Datta, Zili Sideratou, Apostolos Avgeropoulos, Yannis V. Simos, Konstantinos I. Tsamis, Dimitrios Peschos, Ioannis V. Yentekakis, Dimitrios P. Gournis

**Affiliations:** 1 Department of Materials Science and Engineering, University of Ioannina, 45110 Ioannina, Greece; 2 Nanomedicine and Nanobiotechnology Research Group, University of Ioannina, 45110 Ioannina, Greece; 3 Laboratory of Physiology, Department of Medicine, School of Health Sciences, University of Ioannina, 45110 Ioannina, Greece; 4 Institute of Nanoscience and Nanotechnology, NCSR “Demokritos”, Aghia Paraskevi, 15310 Attikis, Greece; 5 Department of Chemistry, Faculty of Engineering and Technology, SRM Institute of Science and Technology, Kattankulathur 603203, Tamil Nadu, India; 6 School of Chemical and Environmental Engineering, Technical University of Crete, 73100 Chania, Greece; 7 Institute of GeoEnergy, Foundation for Research and Technology-Hellas, 73100 Chania, Greece

**Keywords:** porous carbon materials, food waste, freeze drying, biocompatibility

## Abstract

Porous carbon materials from food waste have gained growing interest worldwide for multiple applications due to their natural abundance and the sustainability of the raw materials and the cost-effective synthetic processing. Herein, orange waste-derived porous carbon (OWPC) was developed through a freeze-drying method to prevent the demolition of the original biomass structure and then was pyrolyzed to create a large number of micro, meso and macro pores. The novelty of this work lies in the fact of using the macro-channels of the orange waste in order to create a macroporous network via the freeze-drying method which remains after the pyrolysis steps and creates space for the development of different types of porous in the micro and meso scale in a controlled way. The results showed the successful preparation of a porous carbon material with a high specific surface area of 644 m^2^ g^−1^ without any physical or chemical activation. The material’s cytocompatibility was also investigated against a fibroblast cell line (NIH/3T3 cells). OWPC triggered a mild intracellular reactive oxygen species production without initiating apoptosis or severely affecting cell proliferation and survival. The combination of their physicochemical characteristics and high cytocompatibility renders them promising materials for further use in biomedical and pharmaceutical applications.

## 1. Introduction

Due to their unique properties, porous carbon materials have been used for decades in a wide range of applications [1]. They can be synthesized from a large variety of inexpensive precursors, and they are typically biocompatible. Their exceptional properties such as their low density, high thermal conductivity, mechanical flexibility, and stability [2,3] make these materials suitable for applications among others in thermal insulation, water purification, organic compound or gas sorbents, catalyst supports, energy conversion [4,5,6,7,8] and in the case of porous carbons with large pore volumes they can also be used as sensor substrates [9]. As for the interdisciplinary field of biomedicine, porous carbon, especially mesoporous carbon, and activated carbon materials have been used for drug delivery applications, tissue engineering, and gene transformation [10].

The synthesis of the aforementioned materials from food waste has recently gained great interest as it adds value to this waste and also provides various environmental benefits. The billion tons per year of waste biomass that end up in landfills from households is a low-cost and sustainable source of carbon to obtain bio-derived carbonaceous materials [11,12]. Furthermore, its structure consists of a network of internal channels for the transportation of water and nutrition ingredients, which can contribute to a stable macroporous and mesoporous structural framework. In addition, it has many trace elements such as nitrogen, oxygen, boron, and sulfur, which can be doped into the carbon network as heteroatoms [13].

The usage of biomass is also beneficial for the environment because the large amount of food that ends up in landfills, which contributes to greenhouse gas emissions (GHGs) with a percentage of approximately 8–10%, can be reduced [14]. Pomegranate peels, coconut shells, grass, watermelon peel, blueberry peel, etc., are only a few examples of the precursors used widely in the bibliography [15,16,17,18]. As for the synthetic processes, the drying or hydrothermal method and template method are the most common procedures in the literature; however, chemical or physical activation is necessary in order to reach high values of pore volume and specific surface area [19,20,21].

In this work, porous carbon from a biomass was fabricated, and its biocompatibility was tested for potential biomedical applications. Waste orange peels were chosen because of their large consumption worldwide from households and from juice, jam, and marmalade production industries. They consist of organic acids and flavonoids in higher concentrations than the edible part, while they have high concentrations of pesticides, cellulose, and hemicellulose [22]. These peels were treated with the freeze-drying method before carbonization at high temperatures. Removing water content of the orange peel waste by liquid nitrogen shock and subsequent freeze-drying could utilize the interior channels as stable porous frameworks during the carbonization process, to develop porous carbons with high surface areas and structural integrity [13]. Notably, the high surface areas created from the waste channels will be further used as potential porous scaffolds for multiple biomedical applications without any need for cost-effective physical or chemical activation.

## 2. Results

The OWPC’s physicochemical and structural properties were studied using a combination of characterization techniques, such as X-ray diffraction (XRD), Raman spectroscopy, FTIR spectroscopy, N_2_ porosimetry, and scanning electron microscopy (SEM).

**Figure 1:**
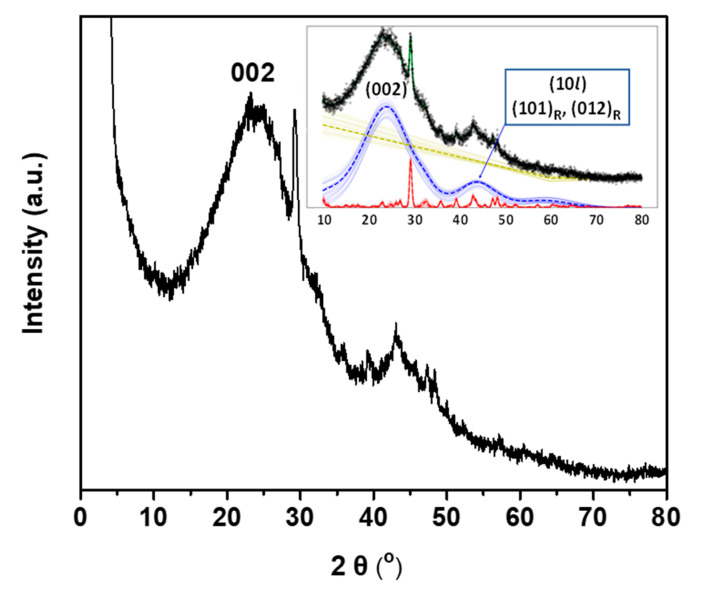
X-ray diffractogram of OWPC. The reflections corresponding to the rhombohedral graphite phase are marked with the subscript ‘R’. The dashed dark yellow line represents the baseline, whereas the dashed red and blue lines represent the crystalline and amorphous phases, respectively.

The X-ray diffraction analysis of OWPC was performed by fitting its diffractogram (Figure 1) using the AMORPH fitting program [23]. The fitting revealed that the amorphous carbon phase constitutes approximately 92% of the total crystallographic composition, while the remainder is crystalline impurities. The amorphous phase, represented by the dashed blue line, reveals an intense and a broad (002) reflection at 2θ = 15–35° characteristic of layered graphitic materials with reasonable periodicity along the [001] direction [24,25]. The weaker and broad reflex at 40–50° can be attributed to a convolution of the (100) and (101) reflections characteristic of the typical hexagonal graphite phase, possibly coming from the (101) and (012) reflections originating from the rhombohedral graphite phase [26]. The crystalline phase, represented by the dashed red line, reveals the characteristic diffraction peaks of the calcite phase (CaCO_3_) (Figure S4). There is a possibility of a potassium calcium phosphate phase (Figure S5), which agrees with the EDXS analysis.

**Figure 2:**
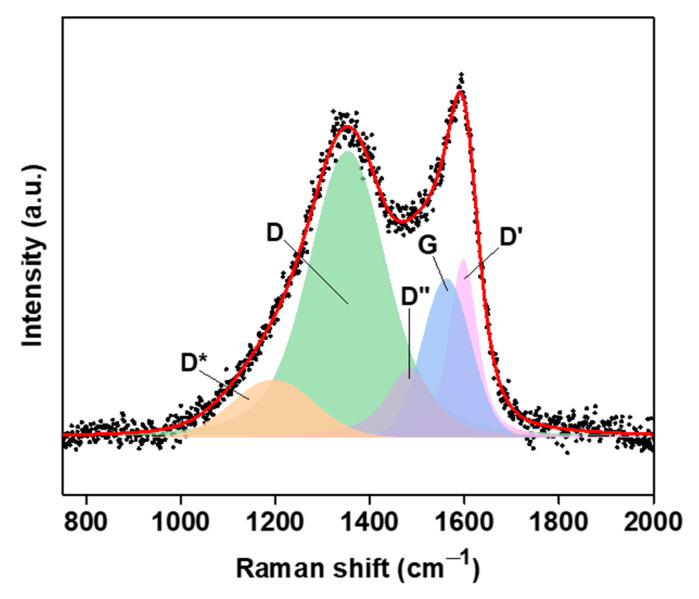
Deconvoluted Raman spectra of OWPC.

Figure 2 shows the deconvoluted Raman spectrum of the OWPC. The spectrum was deconvoluted by fitting it to five symmetric Voigt-type peaks corresponding to the D*, D, D″, G, and D′ bands characteristic of graphitic nanomaterials with defects [27,28]. The curve fitting parameters are presented in Table 1. The spectrum reveals an intense and a broad D band, which corresponds to abundant structural defects distorting the graphitic backbone of the OWPC. These defects can be in the forms of boundary-like defects (weak defects), vacancy-like defects (intermediate defects), and/or bond-angle disorder in *sp*^3^-hybridized and strained *sp*^2^-hybridized domains (strong defects) [29]. The G peak originates from the C-C stretching vibrations of the *sp*^2^-hybridized carbon atoms present in chains and graphitic domains [28]. It is well established that the D-to-G and D′-to-G peak intensity ratios are valuable parameters for quantifying the level of defects in graphene-based materials [30,31]. However, it is worth noting that in the case of OWPC, the contribution of the non-graphitic phase, the *sp*^2^-hybridized carbon chains specifically, to the G band should also be considered. Moreover, these band intensity ratios do not consider the effect of peak broadening [28], thereby diminishing the vital contribution of the bond–angle disorder [32]. For this reason, we propose the D-to-G and D′-to-G integrated intensity ratios ($I_{\mathrm{int},\;D}/I_{\mathrm{int},\;G}$ and $I_{\mathrm{int},\;D^{\prime }}/I_{\mathrm{int},\;G}$, respectively) as alternative reliable parameters that are directly proportional to the concentration and number of defects. The calculated $I_{\mathrm{int},\;D}/I_{\mathrm{int},\;G}$ and $I_{\mathrm{int},\;D^{\prime }}/I_{\mathrm{int},\;G}$ values for the OWPC are 3.32 and 0.81, respectively.

**Figure 3:**
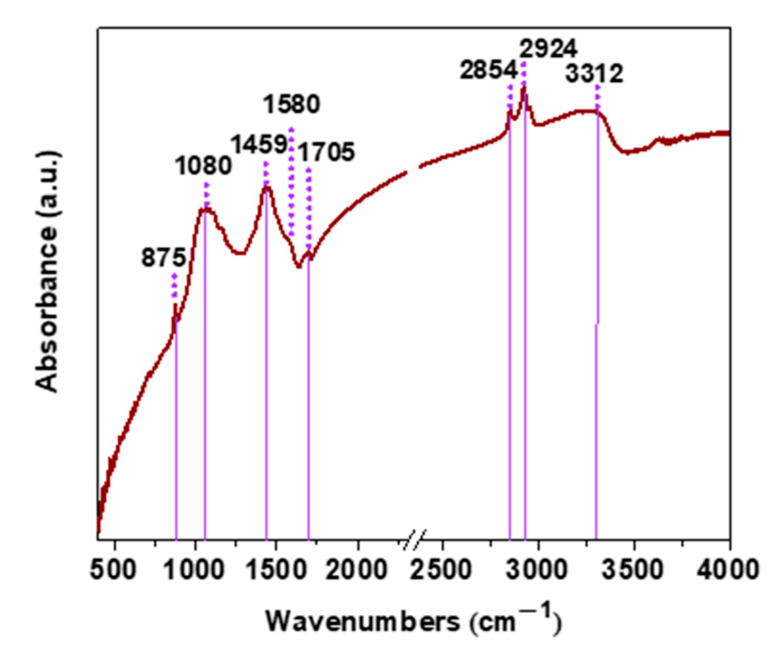
FTIR spectrum of OWPC.

FTIR measurement was carried out to confirm the presence of functional groups in the structure of the OWPC material (Figure 3). The infrared spectrum, in the range of 800–1800 cm^−1^, reveals the existence of multiple peaks. More specifically, the band at 875 cm^−1^ is attributed to the wagging vibrations of hydroxyl groups, while the bands at 1080 cm^−1^ and 1459 cm^−1^ to the stretching vibrations of C-O-C groups and the O-H deformation in the plane, respectively [33,34]. It is worth noting that the aforementioned peaks may derive from calcium carbonate vibration bands. However, due to overlapping with the carbon functionalities of the main carbon frame of the material, it is not clear enough to distinguish. In detail, the peak which is centered at 875 cm^−1^ (ν_2_) corresponds to the out-of-plane bending absorption, at 1080 cm^−1^ (ν_1_) to the carbonate ion symmetric stretching and at 1459 cm^−1^ (ν_3_) to the asymmetric stretching [35,36]. The appearance of the peaks at 1580 cm^−1^ and 1705 cm^−1^ are assigned to the vibrations of C=C bonds of the graphitic domain and to the stretching vibrations of C=O of carboxyl/carbonyl groups, respectively [37]. The bands at 2854 cm^−1^ and 2924 cm^−1^ correspond to the stretching vibrations of alkyl groups [38,39]. Finally, due to the hydrophilic character of the material, the peak at 3312 cm^−1^ is ascribed to the stretching vibrations of the O-H groups of the absorbed water molecules [40].

**Figure 4:**
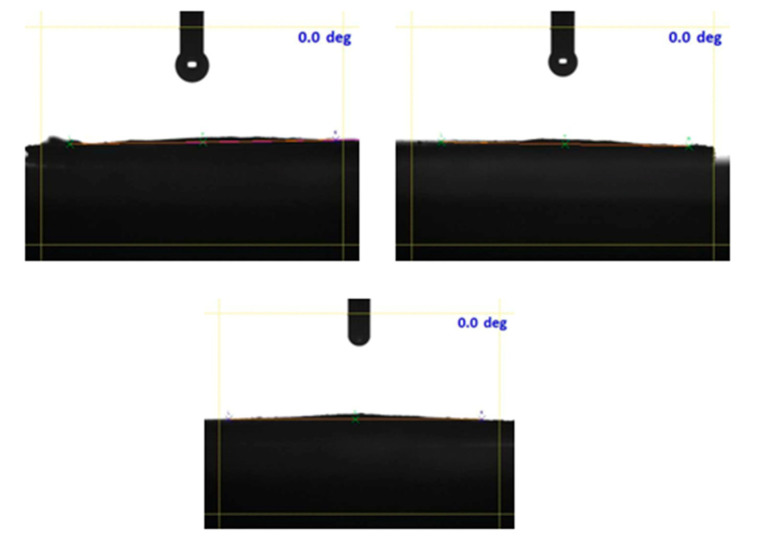
Water contact angle measurements on OWPC.

The functional groups present in the carbon network are further supplemented by water contact angle (WCA) analysis. Upon addition of a water droplet over the OWPC, water wets the surface immediately with a contact angle of 0° implying the superhydrophilicity of the sample (Figure 4).

**Figure 5:**
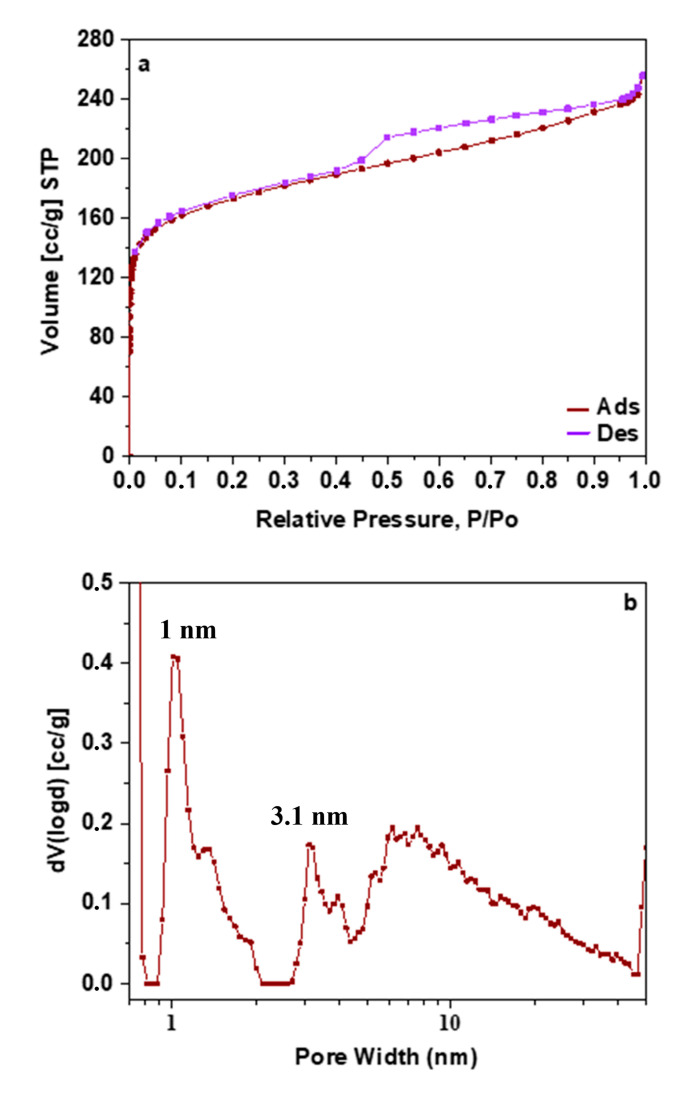
(**a**) BET-nitrogen adsorption/desorption isotherm; and (**b**) Pore size distribution of OWPC material.

Nitrogen porosimetry measurement at −196 °C was further studied to evaluate the textural characteristics. In Figure 5a, the N_2_ adsorption–desorption isotherm and in Figure 5b the Density Functional Theory (DFT) pore size distribution are presented. Based on the IUPAC classification, the N_2_ adsorption isotherm is relative to a type IV material with an H4 hysteresis loop [41]. The textural characteristics of the OWPC are listed in Table 2. The OWPC displays a surface area (S_BET_) of 644 m^2^/g, and the total micro and meso pore volume is calculated at 0.4 cm^3^/g. The DFT model was applied to evaluate the pore size distribution. It was found that the OWPC mainly consists of two different pore sizes (1 nm and 3.1 nm).

**Figure 6:**
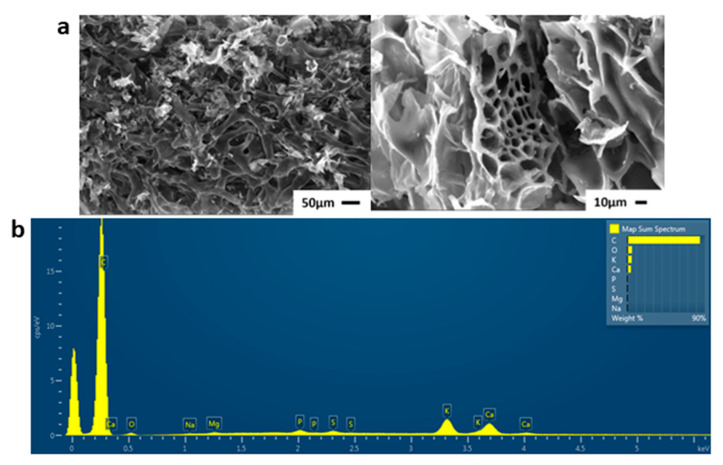
(**a**) SEM images of OWPC, (**b**) EDS elemental quantitative analysis spectrum.

Scanning electron microscopy (SEM) is a powerful tool used to investigate the structure, morphology, and the formation of the material. The morphological characteristics of OWPC were evaluated by SEM images (Figure 6a), showing that the fabricated samples have a self-ordered, uniform, and high-density with homogeneous distribution morphology of macropores throughout the surface. Moreover, the original macropore structure from the channels is well preserved. Energy Dispersive Spectroscopy-EDS spectra of elemental and mapping analysis was performed (Figures S1–S3 and Table S1), which shows the chemical analysis that identified typical elements such as the excess presence of carbon (84.38 wt%) in comparison with oxygen (5.27 wt%) and other elements such as potassium (4.93 wt%), calcium (4 wt%), sodium (0.12 wt%), magnesium (0.25 wt%), sulfur (0.46 wt%), and phosphorus (0.6 wt%) (Figure 6b).

**Figure 7:**
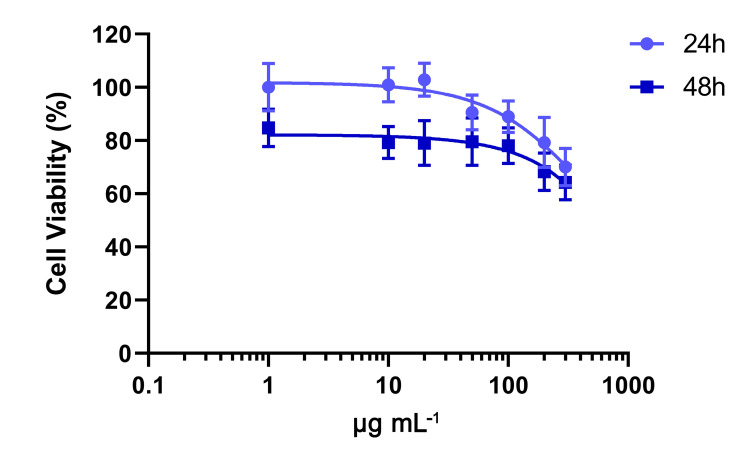
Viability of NIH/3T3 cells after exposure to increasing concentration of OWPC for 24 and 48 h as determined by MTT assays.

To establish their cytocompatibility, NIH/3T3 cells were exposed to OWPC for 24 and 48 h, revealing a dose- and time-dependent cytotoxicity. After 24 h of treatment, OWPC demonstrated no toxicity to cells at doses up to 100 μg mL^−1^, maintaining cell viability above 80%. However, viability gradually declined with increasing doses, reaching 70% viability at 300 μg mL^−1^. Prolonged exposure to OWPC (for 48 h) resulted in decreased cell viability rates, particularly at doses lower than 100 μg mL^−1^, where a drop of 15–20% was observed. At the highest concentration of 300 μg mL^−1^, cell viability was estimated to be 64.2% ± 6.5% (Figure 7).

**Figure 8:**
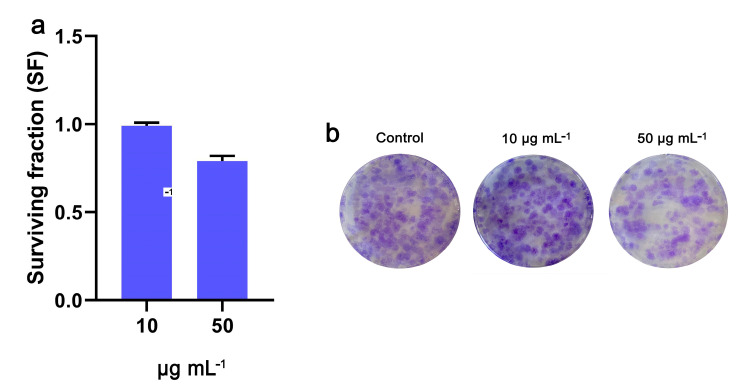
The long-term effect of OWPC on NIH/3T3 cells’ ability to form colonies (**a**). Representative images of colonies formed after incubation with 10 and 50 μg mL^−1^ of OWPC for 24 h (**b**).

Long-term survival, assessed via a clonogenic assay, of NIH/3T3 cells following treatment with OWPC appears to correlate with the findings obtained from the cell viability study. The cells exhibited proliferation and colony formation rates consistent with those observed in the MTT assay. Specifically, the survival fraction (SF) at 10 μg mL^−1^ was equivalent to that of control cells (0.99) and decreased to 0.79 after exposure to 50 μg mL^−1^ (Figure 8), indicating that OWPC did not induce irreversible damage to the cells that would compromise their long-term survival.

**Figure 9:**
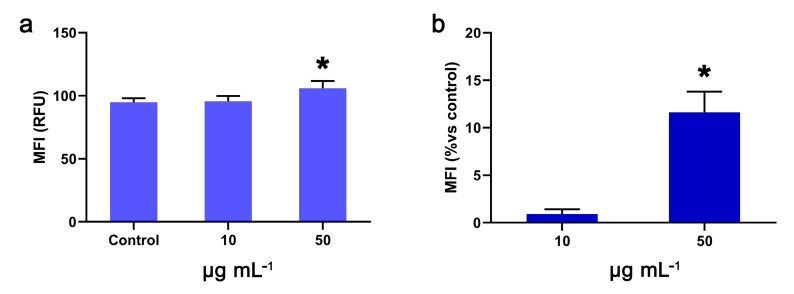
Intracellular ROS formation after exposure to 10 and 50 μg mL^−1^ OWPC for 24 h. Mean fluorescence intensity (MFI) in NIH/3T3 cells (**a**). Increase in MFI versus control (**b**). *, statistically significant difference from control (*p* < 0.05).

OWPC led to the mild formation of ROS in NIH/3T3 cells (Figure 9a). Staining with DCFDA revealed that intracellular ROS increased by 11.6% ± 2.2% (*p* < 0.05) after treatment with 50 μg mL^−1^ OWPC for 24 h (Figure 9b).

**Figure 10:**
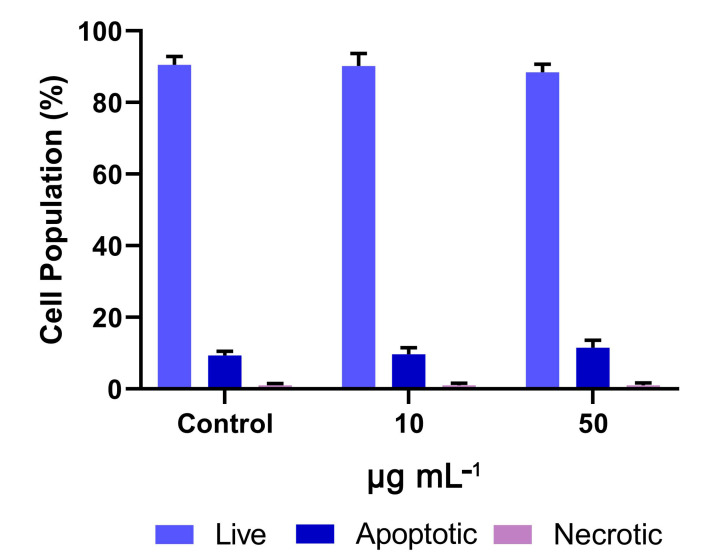
The effect of OWPC on NIH/3T3 cell population.

The population of cells undergoing apoptosis after treatment with 50 μg mL^−1^ OWPC was estimated at 11.5% ± 2.1%, which was similar to that of the control cells (9.4% ± 1.2%) (Figure 10).

## 3. Discussion

According to the results, OWPC material was successfully synthesized through a low-cost route, preventing the deformation of the interior channels, which led to a highly specific area material. The SEM images confirmed the preserved original macroporous structure of the biomass, while the BET measurements revealed the formation of microscale and mesoscale pores through the pyrolysis stage. The SSA was found to be 644 m^2^ g^−1^, which is one of the highest values in the bibliography [13,42,43,44], even without any chemical or physical activation [45]. Moreover, EDX analysis showed that the elements, such as potassium, calcium, etc., were maintained on the final produced material.

As for the cytocompatibility study, data on the interaction between cells and bio-derived carbon porous materials are limited. Alothaid et al. investigated the biological activity of commercially available AC (CAC), pharmaceutical AC (PAC), and AC from date palm kernels (AAC). Human colorectal cancer cells (HCT-116) and liver cancer cells (HepG2) were exposed to increased concentrations of AC for 24 h. The authors showed that all three AC materials exerted a dose-dependent cytotoxicity to both cell lines, with IC50 values ranging from 48.7 ± 17.2 μg mL^−1^ to 88.5 ± 8.9 μg mL^−1^ [46]. Compared with the results in this work, OWPC was less toxic with IC50 values higher than 300 μg mL^−1^. Magno et al. showed that carbon-porous microparticles with a mean size of 250 nm could penetrate HEK293 (human embryonic kidney cells), and HeLa (cervical cancer) cell membranes in low numbers, whereas particles with a size of 662 nm were mainly seen in the bulk solution or around the cell membrane. However, these particles and OWPC can cross cell membranes more effectively in the presence of a transfection agent, such as FugGENE, which ensures high internalization primarily in the cytoplasm and perinuclear region [47]. Moving on to studies on the cytotoxicity of non-bio-derived carbon porous materials, Saha et al. (2016) have shown that highly porous commercial carbon with a very high surface area exhibited greater toxicity against CaCo-2 cells (colorectal adenocarcinoma) compared to mesoporous carbon with a low surface area and large pore width. Interestingly, the latter carbon materials were non-toxic to CaCo-2 cells at doses up to 300 μg mL^−1^ after five days of incubation, whereas the former induced a 30% reduction in the cell population [48]. None of the carbon samples at concentrations as high as 300 μg mL^−1^ demonstrated statistically significant ROS generation, which contradicts our results showing that carbon materials with micro, meso, and macro pores induced ROS formation. Consistent with our findings, a hierarchical nanoporous carbon (HNC) material with uniform particles (mean diameter ~150 nm) and a high specific surface area of 1000 m^2^/g exhibited toxicity against the human glioblastoma cell line U87, leading to a >40% reduction in cell viability after 24 h of incubation with 100 μg mL^−1^ of HNC. The analysis also showed that these nanoparticles induced excessive ROS generation, leading to apoptosis and consequently cell death [49]. Lastly, exposure of AV79 cells (Chinese hamster lung, male) to high doses (10 mg mL^−1^) of novel activated carbon adsorbents (MAST Carbon International Ltd., Basingstoke, UK) with an approximate mean mesopore/macropore size of 19–27 nm did not cause changes in colony numbers and appeared to be non-toxic to the V79 cell line [50].

## 4. Materials and Methods

### 4.1. General Experimental Details

Merlin oranges from Chania, Crete, were purchased from a grocery store in Ioannina, Greece. Dulbecco’s Modified Eagle’s Medium High glucose, Phosphate Buffer Saline (PBS), Thiazolyl blue tetrazolium bromide (MTT), 2′,7′-Dichlorofluorescin diacetate, ≥97% (DCFDA), and Crystal Violet were obtained from Sigma-Aldrich Chemical Co. (St. Louis, MO, USA). Fetal bovine serum (FBS) was purchased from PAN BIOTECH (Aidenbach, Germany). Trypsin-EDTA, Penicillin-Streptomycin, and L-Glutamine were purchased from Biowest (Riverside, CA, USA). Hanks’ Balanced Salt Solution (HBSS) was obtained from Biosera (Nuaille, France). Glutaraldehyde 25% and Dimethyl sulfoxide (DMSO) were purchased from Thermo Fisher Scientific Pharmaceutics Inc. (Waltham, MA, USA). FITC Annexin V, Annexin V Binding buffer and Propidium Iodide (PI) were purchased from BioLegend Inc. (San Diego, CA, USA).

### 4.2. Preparation of the Porous Carbon from Orange Peels

**Figure 11:**
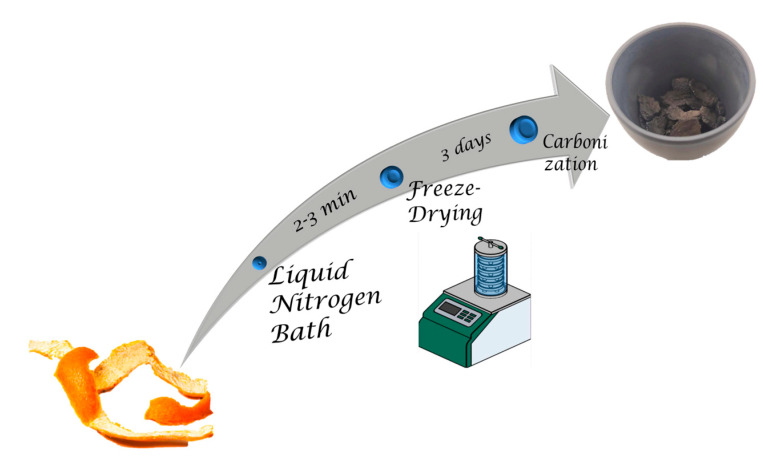
Schematic representation of the porous carbon from orange peels synthetic procedure.

Orange peels were frozen in liquid nitrogen for a couple of minutes and then they were freeze-dried for 3 days. After the freeze-drying stage, 3 g of the material was carbonized under vacuum with a rate of 5 °C/min till the temperature reached the value of 900 °C and remained at this temperature for 2 h (Figure 11). Finally, the material was pulverized. The carbonization yield was estimated to be approximately 50%.

### 4.3. Material Characterization

The XRD pattern was measured on a D8 Advance Bruker (Bruker Corporation, Billerica, MA, USA) powder X-ray diffractometer equipped with graphite-monochromatized Cu Kα radiation (λ = 1.54 Å) from 2° to 80°. Raman spectra were recorded with a Micro-Raman system, RM 1000 RENISHAW (RENISHAW plc, Wotton-under-Edge, Gloucestershire, UK), using a laser excitation line at 532 nm (Nd–YAG), in the range of 1000–3500 cm^−1^. A power level of 1 mW was utilized with a 1 µm focusing spot to avoid the photodecomposition of the samples. FTIR spectrum was collected with a Perkin–Elmer Spectrum GX infrared spectrometer (Perkin–Elmer, Inc., Shelton, CT, USA) featuring a deuterated triglycine sulphate (DTGS) detector. Every spectrum was the average of 64 scans taken with 2 cm^−1^ resolution. The sample was prepared as KBr pellet with ca. 2 wt% of the sample. The SSA and pore distributions were determined by Brunauer–Emmett–Teller (BET) and DFT methods, respectively, from N_2_ adsorption–desorption isotherms at −196 °C from an Autosorb iQ gas sorption analyzer of Quantachrome Instruments (Anton Paar QuantaTec Inc., Boynton Beach, FL, USA). Before the measurement, the samples were degassed for 12 h in a vacuum of 10^−4^ mbar at 120 °C. The relative pressure P/Po was in the range of 0.01–0.30, and the volume of N_2_ sorbed at the highest relative pressure. Scanning electron microscopy (SEM) images were obtained using a JEOL JSM-6510 LV SEM Microscope (JEOL Ltd., Tokyo, Japan) equipped with an X–Act EDS-detector by Oxford Instruments, Abingdon, Oxfordshire, UK (an acceleration voltage of 20 kV was applied). Water contact angle measurement for the OWPC sample was performed using KYOWA DMs-40 (Kyowa Electronic Instruments, Tokyo, Japan), half-angle method fitting, and by FAMAS Add-in software with 2 μL water.

### 4.4. Cell Line

NIH/3T3 cells, which are embryo fibroblasts from Swiss Albino Mice (NIH/3T3, ATCC CRL-1658), were used in this study. The cells were cultured in sterile dishes with a diameter of 10 cm using high-glucose DMEM supplemented with 1% (*v*/*v*) penicillin-streptomycin, 1% (*v*/*v*) L-glutamine, and 10% (*v*/*v*) FBS. They were maintained in an incubator at 37 °C with a composition of 95% air and 5% CO_2_.

### 4.5. Cell Viability Assay

To evaluate cell viability, the 3-(4,5-dimethylthiazol-2-yl)-2,5-diphenyltetrazolium bromide (MTT) assay was employed as previously described [38]. Briefly, 5 × 10^3^ cells were seeded in a 96-well plate and incubated for 24 h before the addition of OWPC at concentrations ranging from 1 to 300 μg mL^−1^. The treatment durations were 24 and 48 h. To minimize DMSO toxicity, its concentration remained below 1% (*v*/*v*) at the highest concentrations used (stock solution concentration was at 30 mg mL^−1^). Subsequently, the MTT reagent was added for 3 h, leading to the formation of formazan crystals at the bottom of the microwells. The crystals were then solubilized with DMSO, and the optical density of the viable cells was measured using a microplate spectrophotometer (Infinite 200 Pro, Tecan, Männedorf, Switzerland). Each set of conditions was replicated three times.

### 4.6. Ability of Cells to Form Colonies (Clonogenic Assay)

The protocol proposed by Franken et al. was followed [51]. NIH/3T3 cells were seeded in 6-well plates (10^3^ cells per well) and allowed to attach for 24 h. Subsequently, cells were treated with 10 and 50 μg mL^−1^ of OWPC for 24 h. After treatment, the supernatant was removed, and fresh medium was added. The cells were cultured for 8 days with a medium change performed on day 4. On the 8th day, the medium was aspirated, and the cells were washed with PBS before staining with 1 mL of dye mixture containing crystal violet (0.5% *w*/*v*) and glutaraldehyde (6% *v*/*v*) for 30 min. Following staining, the excess of dye was removed by rinsing the plates and air-drying at room temperature (25 °C). The quantification of visibly stained colonies was performed using Open CFU open-source software (version 3.9.0) [52], and the surviving fraction (SF%) of the cells was then determined. All experiments were repeated three times.

### 4.7. Flow Cytometry

#### 4.7.1. Reactive Oxygen Species Formation

NIH/3T3 cells (150 × 10^3^ cells per well) were cultured in 6-well plates. Following cell attachment to the plates, OWPC was added at concentrations of 10 and 50 μg mL^−1^ for 24 h. Subsequently, cells were washed with PBS, detached with trypsin, centrifuged at 500× *g* for 5 min, and re-suspended in 2 mL of HBSS containing 2.5 µM DCFDA for 30 min at 37 °C in the dark. The samples were stained with PI (1 μg mL^−1^), chilled on ice, and directly analysed using a fluorescence-activated cell sorting flow cytometer (Partec ML, Partec GmbH, Leipzig, Germany). 10^4^ events were measured for each sample, and all experiments were repeated three times.

#### 4.7.2. Detection of Apoptosis

The NIH/3T3 cells (5 × 10^4^ cells per well) were seeded in 48-well plates and incubated for 24 h before being treated with 10 and 50 μg mL^−1^ of OWPC for an additional 24 h. Cells were then detached and counted with a Neubauer hemocytometer, and 10^5^ cells from each well were transferred to a clean Eppendorf tube, centrifuged (500× *g*), re-suspended in 100 µL of Annexin V Binding buffer and stained with FITC Annexin V and PI for 15 min at room temperature in the dark. Following the addition of 400 µL of Annexin V binding buffer, the samples were analyzed using a flow cytometer (Partec ML, Partec GmbH, Leipzig, Germany). All experiments were replicated in triplicate.

## 5. Conclusions

In conclusion, orange waste derived porous carbon was synthesized and fully characterized. The natural abundance and sustainability of the orange waste as a precursor, as well as the cost-effective synthetic procedure, make this material attractive for a wide variety of applications. More specifically, the porous material was developed via a synthetic process which allows us to use the biomass original structural framework as the meso and macro porous structure of the material. The micro porous structure of the material was increased through the carbonization stage. OWPC exerted mild toxicity to normal cells potentially through the generation of ROS. Nonetheless, their high surface area, without any chemical or physical activation, and hydrophilic profile classify them as a promising platform for further exploration and potential modification for biomedical applications such as drug delivery, biosensing, and bioimaging.

## Figures and Tables

**Table 1:** Curve fitting parameters for the deconvoluted Raman spectrum of OWPC. $\Delta \tilde{\nu }$ = Raman shift/band position, and $I_{int}$ = Integrated intensity.

Parameters	Raman Bands				
	D*	D	D″	G	D′
$\Delta \tilde{\nu }$	1197 cm^−1^	1354 cm^−1^	1486 cm^−1^	1564 cm^−1^	1600 cm^−1^
$I_{int}$	26,796 cm^−1^	147,808 cm^−1^	26,026 cm^−1^	44,473 cm^−1^	35,818 cm^−1^

**Table 2:** Textural characteristics of OWPC.

BET SSA (m^2^/g)	Total Pore Volume (cm^3^/g)	Micropore Volume (cm^3^/g)	Mesopore Volume (cm^3^/g)
644	0.4	0.2	0.2

## Data Availability

Data are available on request due to restrictions.

## Supplementary Materials

The following supporting information can be downloaded at: https://www.mdpi.com/article/10.3390/molecules29163967/s1, Figure S1: EDS Elemental mapping of OWPC; Figure S2: EDS Element mapping of Mg and Na, respectively; Figure S3: EDS Element mapping of C, Ca, K, S, P, O, respectively; Table S1: EDS elemental quantitative analysis; Figure S4: XRD fitting of Calcite phase; Figure S5: XRD fitting of Calcite and Potassium Calcium Phosphate phase.
