# Supplementary material for: A Strategic Synthesis of Orange Waste-Derived Porous Carbon via a Freeze-Drying Method: Morphological Characterization and Cytocompatibility Evaluation
Source: Molecules. 2024 Aug 22;29(16):3967. doi: 10.3390/molecules29163967 (PMC11357121; doi:10.3390/molecules29163967)
Supplement: Supplementary file 1 [file molecules-29-03967-s001.zip › molecules-3117133-supplementary.pdf]

Supporting information

A strategic synthesis of orange waste-derived porous carbon via Freeze-drying method: Morphological characterization and Cytocompatibility evaluation

Angela S. Kaloudi [1], Panagiota Zygouri [1,2], Konstantinos Spyrou [1,2*], Antrea-Maria Athinodorou [2,3], Eirini Papanikolaou [2,3], Mohammed Subrati [4], Dimitrios Moschovas [1], K.K.R. Datta [5], Zili Sideratou [4], Apostolos Avgeropoulos [1], Yannis V. Simos [2,3], Konstantinos I. Tsamis [2,3], Dimitrios Peschos [2,3], Ioannis V. Yentekakis [6,7], Dimitrios P. Gournis [6,7]

[1] Department of Materials Science and Engineering, University of Ioannina, 45110 Ioannina, Greece; a.kaloudi@uoi.gr (A.S.K.); pzygouri@gmail.com (P.Z.); konstantinos.spyrou1@gmail.com (K.S.); dmoschov@uoi.gr (D.M.); aavger@uoi.gr (A.A.)

[2] Nanomedicine and Nanobiotechnology Research Group, University of Ioannina, Ioannina 45110, Greece; pzygouri@gmail.com (P.Z.); konstantinos.spyrou1@gmail.com (K.S.); amathinodorou@yahoo.com (A.-M.A.); epapanikolaou@uoi.gr (E.P.); isimos@uoi.gr (Y.V.S.); ktsamis@uoi.gr (K.I.T.); dpeschos@uoi.gr (D.P.)

[3] Laboratory of Physiology, Department of Medicine, School of Health Sciences, University of Ioannina, 45110 Ioannina, Greece; amathinodorou@yahoo.com (A.-M.A.); epapanikolaou@uoi.gr (E.P.); isimos@uoi.gr (Y.V.S.); ktsamis@uoi.gr (K.I.T.); dpeschos@uoi.gr (D.P.)

[4] Institute of Nanoscience and Nanotechnology, NCSR "Demokritos", 15310, Aghia Paraskevi, Attikis, Greece; mossubrati@gmail.com (M.S.); z.sideratou@inn.demokritos.gr (Z.S.)

[5] Department of Chemistry, Faculty of Engineering and Technology, SRM Institute of Science and Technology, Kattankulathur, Tamil Nadu 603203, India; kumarard@srmist.edu.in

[6] School of Chemical and Environmental Engineering, Technical University of Crete, GR-73100 Chania; yyentek@isc.tuc.gr (I.V.Y), dgournis@tuc.gr (D.P.G.)

[7] Institute of GeoEnergy, Foundation for Research and Technology-Hellas, GR-73100 Chania; yyentek@isc.tuc.gr (I.V.Y), dgournis@tuc.gr (D.P.G.)

* Dimitrios P. Gournis: dgournis@tuc.gr; Konstantinos Spyrou: konstantinos.spyrou1@gmail.com

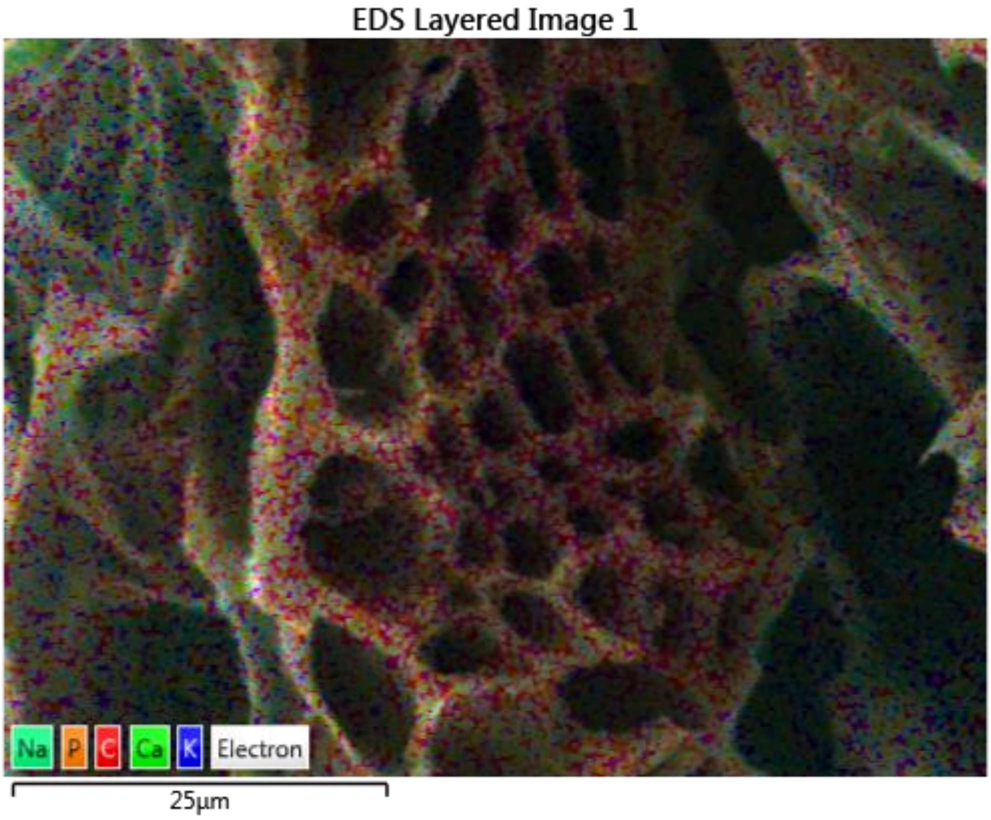

Figure S1. EDS Elemental mapping of OWPC

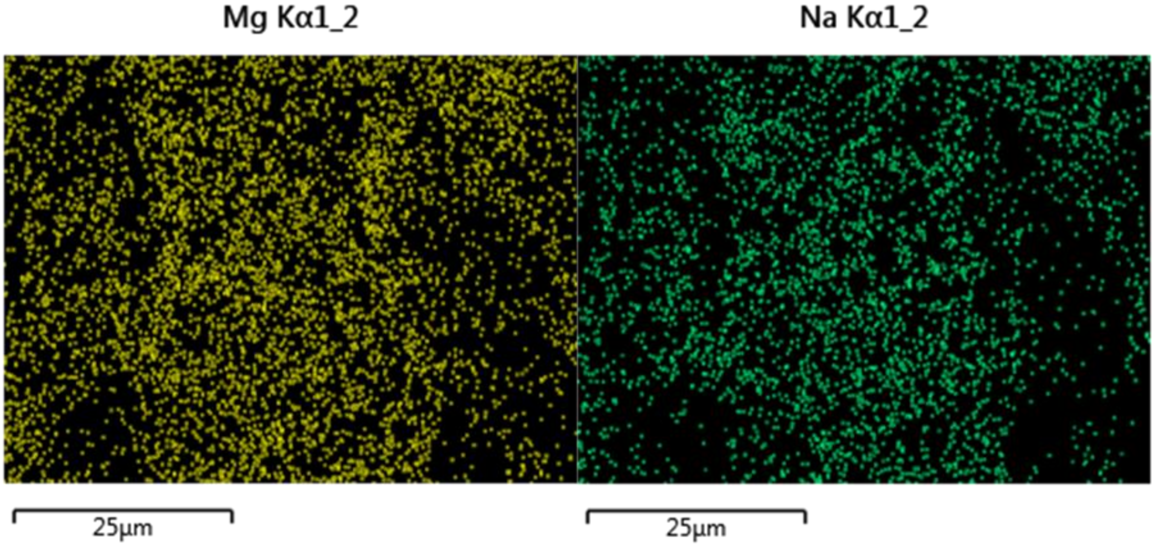

Figure S2. EDS Element mapping of Mg and Na, respectively.

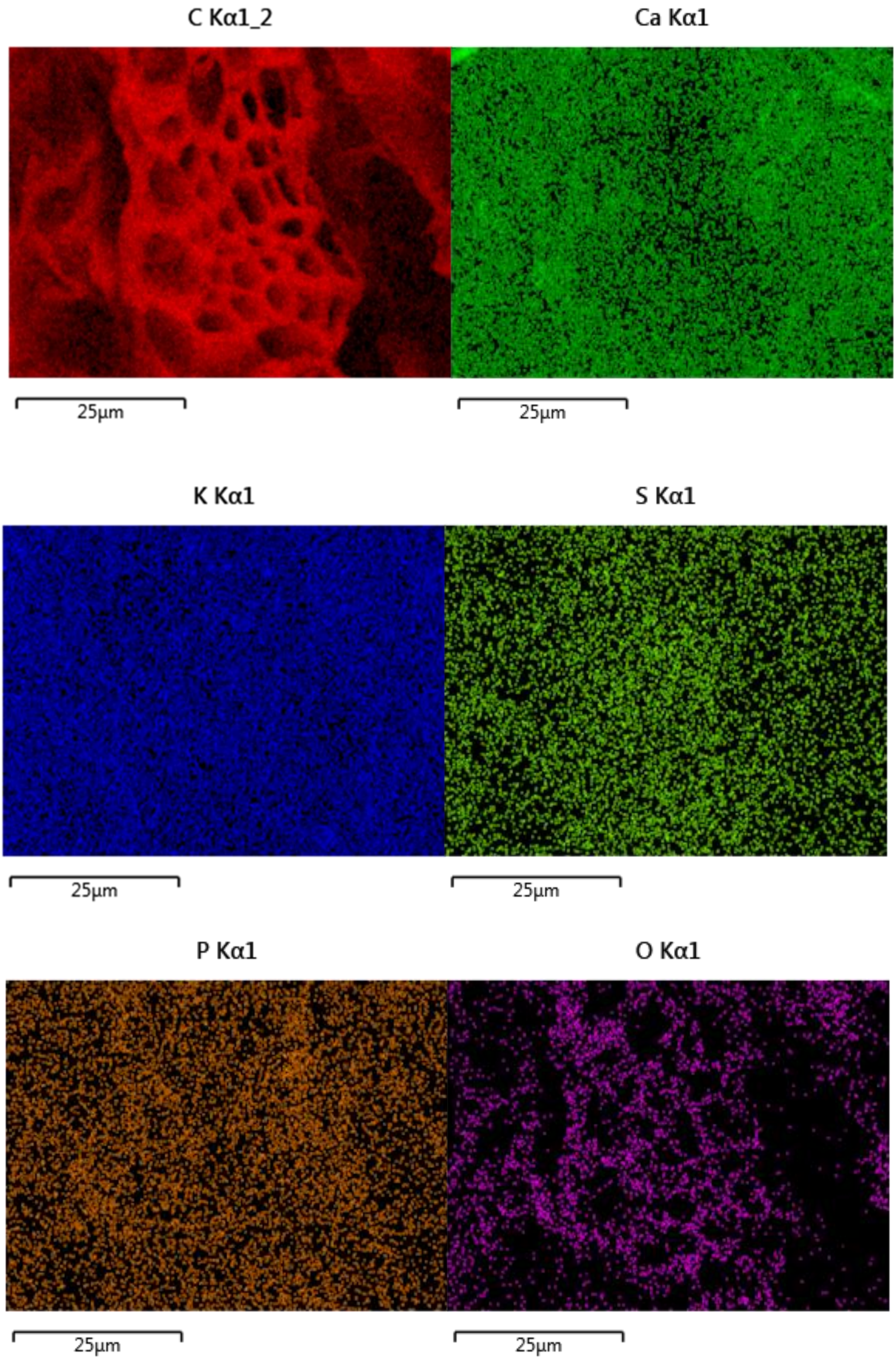

Figure S3. EDS Element mapping of C, Ca, K, S, P, O, respectively.

Table S1. EDS elemental quantitative analysis

Map Sum Spectrum	Wt%	Wt% Sigma
C	84.38	0.22
O	5.27	0.20
Na	0.12	0.03
Mg	0.25	0.02
P	0.60	0.03
S	0.46	0.03
K	4.93	0.06
Ca	4.00	0.06
Total	100.00	

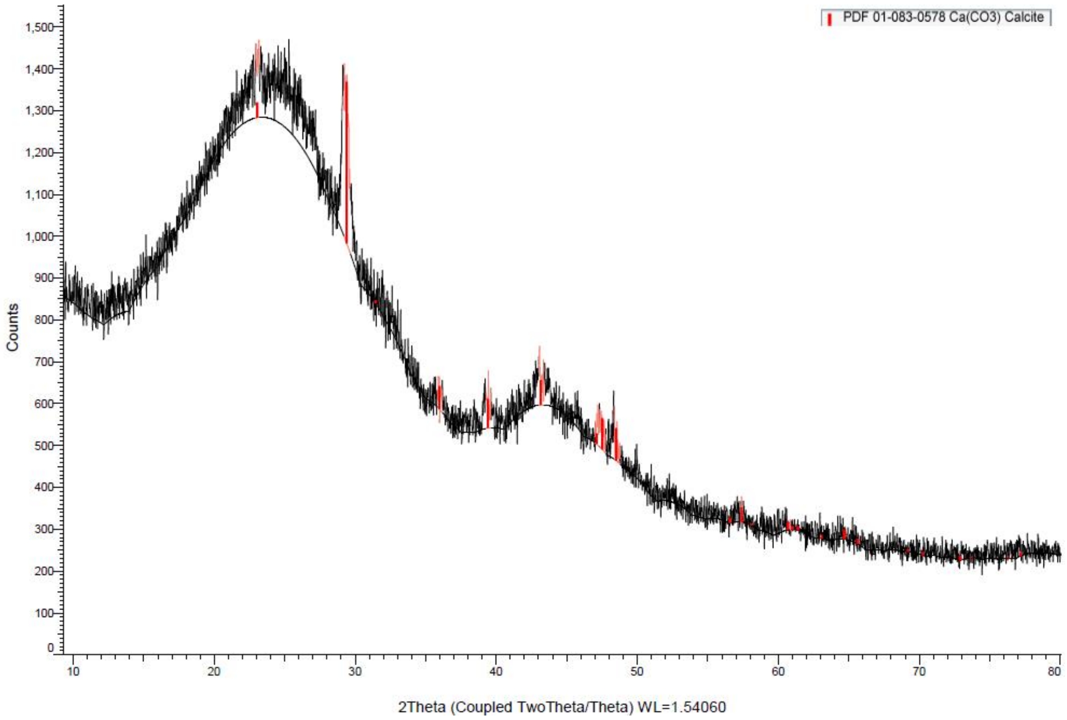

Figure S4. XRD fitting of Calcite phase.

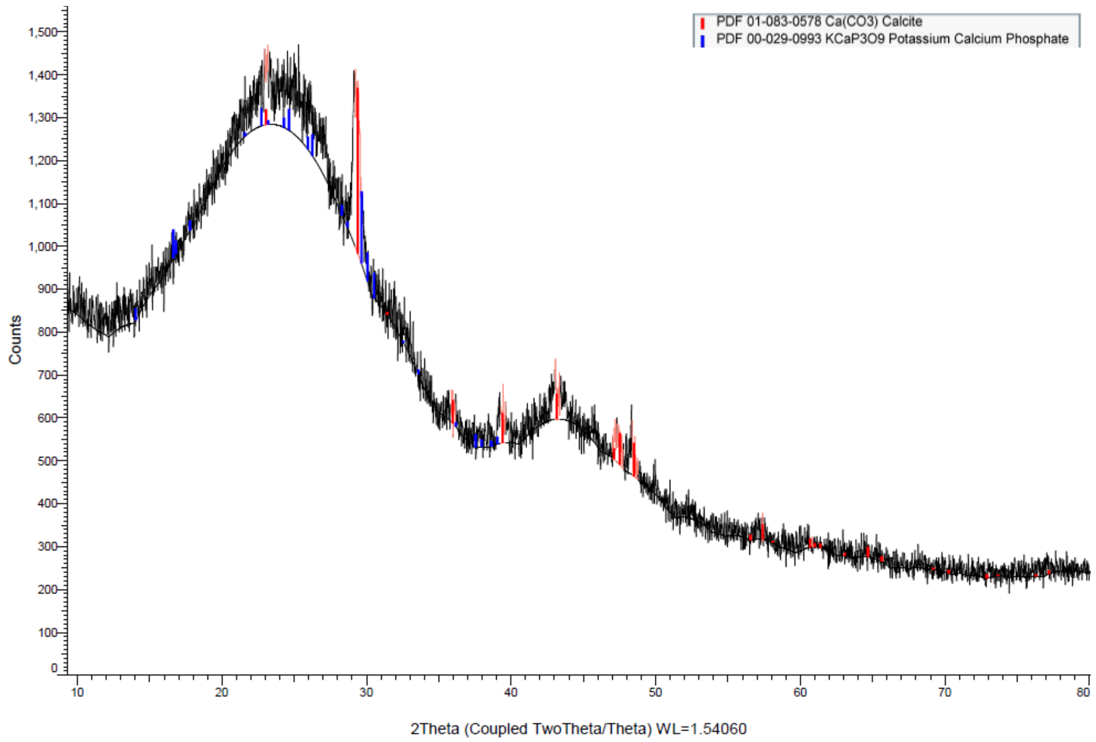

Figure S5. XRD fitting of Calcite and Potassium Calcium Phosphate phase.
